# Preschool Children with High Reading Ability Show Inversion Sensitivity to Words in Environment: An Eye-Tracking Study

**DOI:** 10.3390/jemr18020004

**Published:** 2025-02-28

**Authors:** Yaowen Li, Jing Zhao, Wangmei Chen, Shaoxue Zhang, Wenjing Zhang, Wei Wang, Limin Xu, Shifeng Li, Licheng Xue

**Affiliations:** 1 Jing Hengyi School of Education, Hangzhou Normal University, Hangzhou 311121, China; liyaowen1999@163.com (Y.L.); zhaojing561@126.com (J.Z.);; 2 Zhejiang Philosophy and Social Science Laboratory for Research in Early Development and Childcare, Hangzhou Normal University, Hangzhou 311121, China; 3 Hangzhou Qiantang District Wenqing Primary School, Hangzhou 310020, China; 4 Zhongtai Central Kindergarten, Hangzhou 311121, China; 5 School of Psychology, Northwest Normal University, Lanzhou 730070, China; 6 School of Preschool Education, Hangzhou Polytechnic, Hangzhou 311402, China

**Keywords:** environmental print, inversion sensitivity, preschool, eye tracking, reading, attention

## Abstract

Words in environmental print are exposed to young children before formally learning to read, and attention to these words is linked to their reading ability. Inversion sensitivity, the ability to distinguish between upright and inverted words, is a pivotal milestone in reading development. To further explore the relationship between attention to words in environmental print and early reading development, we examined whether children with varying reading abilities differed in inversion sensitivity to these words. Participants included children with low (18, 8 males, 5.06 years) and high (19, 10 males, 5.00 years) reading levels. Using an eye-tracking technique, we compared children’s attention to upright and inverted words in environmental print and ordinary words during a free-viewing task. In terms of the percentage of fixation duration and fixation count, results showed that children with high reading abilities exhibited inversion sensitivity to words in environmental print, whereas children with low reading abilities did not. Unexpectedly, in terms of first fixation latency, children with low reading abilities showed inversion sensitivity to ordinary words, while children with high reading abilities did not. These findings suggest that inversion sensitivity to words in environmental print is closely linked to early reading ability.

## 1. Introduction

Environmental print, such as the “KFC” on advertising billboards, is the type of print children often encounter before formally learning to read. Attention to words in environmental print is closely linked to children’s reading ability [1,2,3,4,5]. The emergence of inversion sensitivity to visual words, that is, the ability to distinguish between upright and inverted words, marks a milestone in the development of reading and serves as an indicator of reading expertise [6,7,8,9,10]. To further understand the relationship between attention to words in environmental print and early reading development, we investigated differences in inversion sensitivity to words in environmental print among preschool children at various levels of reading ability.

Before children begin formal reading instruction in school, they are already familiar with a wide array of printed texts in their daily environment, termed ‘environmental print’, such as words in traffic signs, trademarks, and advertisements [11,12,13]. Environmental print consists of visual word forms and contextual cues such as color, logo, and art font. The visual word information, namely, words in environmental print, is composed of low-frequency Chinese characters that are not common in children’s lives, but children are more familiar with the words as a whole. Studies indicate that as children’s reading skills progress, they become increasingly proficient at extracting textual information from environmental print texts [5,12,14]. During the school-age learning process, attention to words is also closely associated with children’s reading ability [15,16]. Children at risk of dyslexia often exhibit weaker attention to words, and their reading skills can be improved through attention training [17,18,19]. In Xue’s 2023 study, children were divided into two groups based on their literacy test scores (i.e., high- and low-reading level groups). The study utilized words in environmental print, stroke combinations resembling these words, and other Chinese character strings as experimental materials. The results revealed that only children with high reading skills exhibited significant differences in attention to words in environmental print compared to other Chinese character strings, suggesting heightened sensitivity to the fine-grained aspect of words in environmental print [20].

In addition, previous findings indicate that inversion sensitivity to visual words emerges at an early age, and marks a pivotal milestone in reading development [9,10]. Specifically, children’s perception of the direction and structure of Chinese characters changes with reading ability [9]. Inversion sensitivity to words is considered an index of reading expertise [21,22]. Using a visual perceptual matching task, Zhang and colleagues (2020) found that 4- to 5-year-olds took longer to react to inverted words compared to normal ones, suggesting that children may develop inversion sensitivity to Chinese characters at an early age. Additionally, children’s inversion sensitivity to words is significantly correlated with their reading proficiency [8,10]. Moreover, such correlation persisted even after adjusting for age, underscoring the close link between children’s inversion sensitivity to words and their reading skills [8].

Previous research has highlighted the relationship between preschool children’s inversion sensitivity to words and their reading ability [8,9,10]. It is also important to note that there are three issues warranting further investigation. First, most previous studies focused on inversion sensitivity to ordinary words [8,10]. Environmental print is more ubiquitous and functional in children’s early daily life [5]. However, to the best of our knowledge, few studies have examined inversion sensitivity to words in environmental print. To compare differences in children’s inversion sensitivity between words in environmental print and ordinary words, while minimizing potential confounding effects related to prior reading experience, we selected Chinese characters that preschool children are unlikely to encounter in their daily lives. These characters, referred to as “ordinary words” in this study, served as the control group for words in environmental print. Ordinary words consist of Chinese characters that are not commonly seen in daily life but closely resemble words in environmental print, in terms of shape and number of strokes. The primary distinction between the two types of words lies in children’s greater exposure to the words in environmental print. By comparing the inversion effects of words in environmental print and ordinary words, we sought to examine whether children develop print awareness earlier for words in environmental print, which are more frequently encountered in daily life. Addressing this question enhances our understanding of the role of environmental print experience in early reading development.

Second, during the preschool years, individual differences in reading ability exist even among children of the same age [20,23,24]. Reading ability closely correlates with inversion sensitivity to words. However, to the best of our knowledge, only one study has directly examined how reading ability modulates attention to words in environmental print. While that study focused on the perspective of visual word form features (i.e., coarse versus fine-grained aspect), it did not explore print awareness [20]. To address this gap, we adopted the group design used in Xue’s study but shifted our focus to examining inversion sensitivity from the perspective of print awareness. This approach aims to deepen our understanding of the relationship between attention to words in environmental print and early reading development. Third, most previous studies employed a visual matching task, which measures inversion sensitivity to words in terms of visual recognition rather than attention [9,10]. The visual matching task is an active task, reflecting children’s judgment on word orientation through task-related strategic processing [9,10]. In contrast, a free-viewing task is a passive task that reflects the automatic processing of word orientation. Eye-tracking technology offers a more direct method for measuring attention [16,25]. By employing eye-tracking technology, we can more accurately capture children’s attention allocation while processing various types of words [26,27,28]. Eye-tracking technology enables real-time recording of visual attention as children view words in different orientations, providing a more precise understanding of their perceptual processes [27,29]. Therefore, this study utilizes eye-tracking technology to expand previous research on children’s inversion sensitivity [9,30,31].

**Figure 1:**
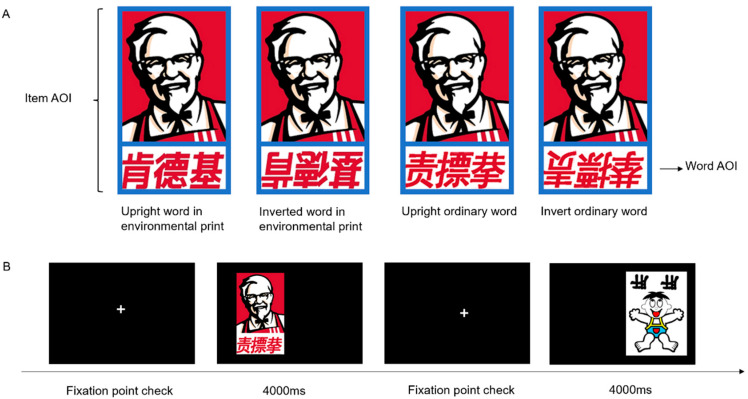
(**A**) Samples of the four versions of stimuli and the division of Areas of Interest (AOI). (**B**) An example of the sequence of events in a mini-block of trials in a free viewing task.

The primary aim of this study was to investigate how exposure to environmental print influences children’s inversion sensitivity to visual words in terms of attention, and its association with their reading ability. Specifically, utilizing eye-tracking technology, we addressed the following questions: (a) Are there differences in children’s inversion sensitivity to words in environmental print versus ordinary words? (b) Do children with high and low reading abilities differ in inversion sensitivity to words in terms of attention? To achieve these objectives, we recruited two groups of preschool children, matched by age but differing in reading ability. The children’s reading abilities were assessed using a widely recognized Chinese word recognition test designed for preschoolers [15,32]. Based on their test scores, the preschool children were divided into high and low reading ability groups using the median segmentation method [20]. To evaluate whether the children can distinguish different types of textual information (i.e., words in environmental print versus ordinary words) in their environment, we preserved the original format of words in environmental print, including contextual cues, such as color, logo, and font type. Ordinary words, which preschoolers are rarely exposed to, were selected and matched with words in environmental print in terms of structure and stroke count. To examine inversion sensitivity, all stimuli were rotated 180 degrees to create four types of word stimuli: (a) upright words in environmental print; (b) inverted words in environmental print; (c) upright ordinary words; and (d) inverted ordinary words. An example of these stimuli is shown in Figure 1A. Using eye-tracking technology, we recorded the children’s eye movements as they freely viewed the pictures.

Drawing on previous research findings [2,9,20,23], we hypothesized that the children’s reading abilities would modulate their inversion sensitivity to visual words in environmental print and ordinary words in terms of attention. Specifically, given the established correlation between inversion sensitivity to words and children’s reading ability [9,10,33], we anticipated that the inverted words would capture more attention from children with high reading ability compared to those with low reading ability. Furthermore, based on the robust relationship between children’s attention to words in their environment and their reading ability [12,15,20], we expected that children with high reading ability would demonstrate greater sensitivity to the inversions of words in environmental print compared to ordinary words.

## 2. Method

The research protocol was meticulously reviewed and approval by the Human Research Review Committee of Hangzhou Normal University, ensuring strict adherence to the ethical principles outlined in the Declaration of Helsinki. Written informed consent for participation was obtained from parents or legal guardians of all the children involved in the study.

## 3. Participants

The sample size for this study was determined using a power analysis conducted with G*Power software [34]. To detect differences using a repeated-measures ANOVA, a total of 36 participants (18 in each group) was required, assuming a power (1 − β) of 0.95, an alpha level of 0.05, and an effect size of 0.25. To enhance the robustness of the results, 40 preschool children were recruited. All participants were native Mandarin speakers with normal or corrected-to-normal vision and had not received formal instruction in reading and writing Chinese characters during kindergarten. Written informed consent was obtained from parents or legal guardians, and oral assent was provided by the children prior to participation. The study protocol was reviewed and approved by the Institutional Review Board of the Institute of Hangzhou Normal University. Each child completed a Chinese word recognition test designed for Hong Kong Chinese preschoolers [32] as no comparable test exists for preschoolers in the Chinese Mainland. This test has been widely used with preschoolers in the Chinese Mainland [5,15,20,35]. The test consists of 61 items, including 27 Chinese characters and 34 words drawn from first-grade Chinese textbooks. The items are arranged in increasing order of difficulty. Children read each item aloud, earning one point per correct response. The total possible scores ranged from 0 to 61 points. Data from one participant were excluded due to over 10% of eye-tracking data loss caused by accidental factors, such as head or body movements. Two additional participants were excluded because they did not complete the Chinese word recognition test. The remaining 37 participants were divided into two groups—low-reading ability and high-reading ability—based on the median score. Table 1 presents demographic characteristics and performance on the Chinese word recognition test. Independent samples *t*-tests confirmed no significant difference in age between the two groups, *t* (35) = 1.028, *p* = 0.311.

## 4. Materials

In this study, we selected 10 well-established environmental print logos that are commonly recognized by Chinese preschoolers, based on previous research [20]. These logos included popular trademarks such as Oreo, Cai Hong, Gong Gong Ce Suo, Hai Di Xiao Zong Dui, Jin Zhi Xi Yan, KFC, Wang Wang, Wang Zai Niu Nai, Peppa Pig, and Xiong Chu Mo. To control for confounding variables, we removed the manufacturer’s name and any additional small printed text, ensuring that only the prominent word in each logo remained visible. For example, on the Skittles logo, we removed the flavors and English text, leaving only the Chinese character “Cai Hong”, along with the background image. To investigate potential differences in children’s attention toward words in environmental print versus ordinary words, as well as the effect of word orientation (upright versus inverted), we created four categories of stimuli: (a) upright words in environmental print; (b) inverted words in environmental print; (c) upright ordinary words; and (d) inverted ordinary words (see Figure 1A for an illustration). Ordinary words were chosen based on their visual similarity to the words in environmental print, considering aspects such as shape and number of strokes. The stroke count was 27.00 ± 6.07 for words in environmental print and 28.60 ± 8.32 for ordinary words. To ensure consistency, all four types of stimuli were presented within the same logo backgrounds. The expected outcome of heightened inversion sensitivity would be observed if the children demonstrated increased attention to the upright words in environmental print. The image were presented at a visual angle of 21.56° × 20.84°, with a viewing distance of 65 cm.

## 5. Apparatus

All stimuli were displayed on a 19-inch LCD monitor, with a fixed viewing distance of 65cm between the child and the screen, maintained by a chin rest. The presentation of the stimuli and recording of responses were managed using a script created with Experiment Builder (SR Research, Ottawa, ON, Canada). An EyeLink 1000 eye tracker (SR Research, Ottawa, ON, Canada) was employed to monitor and record the children’s gaze direction. The eye tracker provided a reported tracking accuracy of 0.25° or better, with a sampling rate set to 1000 Hz.

## 6. Procedure

To assess the children’s attention to words within logos in a natural state, we employed a free-viewing approach, following the methodology used in previous studies [5,20,36] (see Figure 1B). All experimental images were presented against a white background. A total of 80 items were included, with each of the ten logos featuring four versions. Each image was presented once in both the left and right positions, in a random order. The items were organized into 4 mini-blocks, each consisting of 10 trials. The four mini-blocks were presented twice, with breaks allowed between presentations to prevent fatigue. Before the experiment began, a standard 9-point calibration for the eye tracker was performed. Children viewed an animated picture of a cartoon frog at eight corners and the center of the screen. Calibration was complete when stable fixation was achieved at all nine points on the grid (within a 0.5° error), ensuring accurate gaze location estimation. If the calibration failed at any point, the process was repeated. Throughout the experiment, the children’s eyes were kept clean and the calibration was repeated before each block to maintain accurate tracking of their eye movements.

Each mini-block consisted of 10 items, with the images presented in a randomized order. To prevent consecutive presentation of the same type of stimuli (i.e., upright environmental print, inverted environmental print, upright ordinary words, and inverted ordinary words), images were carefully arranged. Following the established protocols, each item was displayed for 4000 ms, and camera calibration was verified after each image presentation. Participants were unaware of the specific purpose of the experiment and were instructed to freely observe the images on the screen. The researcher provided the following instructions: ‘After the little frog, some pictures will appear on the screen, and all you need to do is freely observe the pictures’.

The experiment was conducted in a quiet kindergarten classroom, with the eye-tracking free-viewing task taking approximately 12 min to complete. After the eye-tracking experiment, the children underwent a Chinese word recognition test to assess their reading abilities.

## 7. Data Preprocessing and Analysis

Eye movement data were processed using Data Viewer (SR Research, Ottawa, ON, Canada). To address the impact of blinking, fixation points occurring immediately after blinking were removed. In line with prior research [5,15,20], nearby fixation points were merged using an amplitude threshold of 1.5 degrees. Additionally, fixations lasting less than 50 ms were excluded from the analysis [5]. As a result, the percentage of invalid eye movement data removed was 1.67%.

We first defined areas of interest (AOIs) for the four versions of 10 logos. AOIs were manually delineated around the word and picture regions of all items, as shown in Figure 1A. Specifically, the word AOI was defined as an independent rectangular or polygonal area that closely enveloped all visual word forms, while the item AOI represented the display region occupied by the entire image [5]. Identical word and item AOIs were applied across the four types of stimuli, as the size and shape of the prints were consistent in all versions. Fixations occurring outside the boundaries of the item AOIs were excluded from the analysis.

Similar to previous studies [5,20], we focused on three indicators to examine attention to words: (a) the percentage of fixation duration in word AOIs (i.e., the duration time of fixations in word AOIs divided by the total duration time of fixations in item AOIs, multiplied by 100); (b) the percentage of fixation count in word AOIs (i.e., the number of fixation counts in word AOIs divided by the total number of fixation counts in item AOIs, multiplied by 100); and (c) the latency of the first fixation in word AOIs (i.e., the time from stimulus presentation to the first fixation on word AOIs). By using eye movement data ratios to standardize and normalize the dataset, we aimed to conduct a more focused comparison of variations in total fixation counts and durations across different participants [15,36].

## 8. Result

Eye movement data were analyzed using a 2 × 2 × 2 three-way repeated measures ANOVA, with Reading Group (Low versus High) as a between-subject variable, and Word Direction (Upright and Inverted) and Stimulus Category (Words in Environmental Print and Ordinary Words) as within-subject variables.

**Figure 2:**
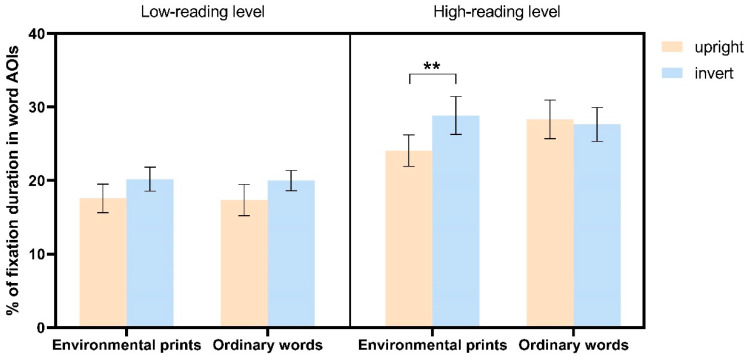
The percentages of fixation duration in word AOIs across four stimulus categories in children at different levels of reading ability. Error bars denote ± standard error of measurement. ** *p* < 0.01.

*The percentages of fixation duration in word AOIs* for the two groups of children are presented in Figure 2. Results revealed a significant main effect of Word Direction (*F* (1, 35) = 5.474, *p* = 0.025, *η*^2^ = 0.135), indicating that the fixation duration in the word AOI was significantly lower for upright images compared to inverted images (*t*(36) = −2.300, *p* = 0.025, *d* = −1.533). Additionally, the two-way interaction of Word Direction by Stimulus Category was significant (*F* (1, 35) = 6.064, *p* = 0.019, *η*^2^ = 0.148). Specifically, for logos with words in environmental print, fixation duration on inverted images was significantly higher than on upright images (*t*(36) = 3.083, *p* = 0.004, *d* = 2.176). In contrast, there were no differences between inverted and upright images for logos with ordinary words (*t*(36) = 0.910, *p* = 0.369, *d* = 0.588). Importantly, the three-way interaction effect among Reading Group, Word Direction, and Stimulus Category was also significant (*F* (1, 35) = 6.196, *p* = 0.018, *η*^2^ = 0.150). As shown in Figure 2, when viewing words in environmental print, children in the high-reading level group showed a higher percentage of fixation duration in word AOIs in the inverted condition than in the upright condition (*t*(18) = 2.824, *p* = 0.007, *d* = 2.400). However, children in the low-reading level group did not show significant inversion sensitivity (*t*(17) = 1.529, *p* = 0.139, *d* = 1.238). Furthermore, there were no significant differences in inversion sensitivity when viewing ordinary words for either the high-reading level or low-reading level groups (all *p*-values > 0.05).

**Figure 3:**
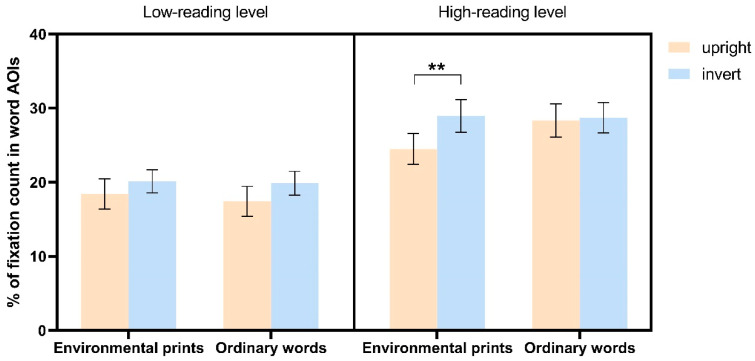
The fixation count percentages within word AOIs across four stimulus categories in children at different levels of reading ability. Error bars denote ± standard error of measurement. ** *p* < 0.01.

*The fixation count percentages within word AOIs* for the two groups of children are presented in Figure 3. The results revealed a significant main effect of Word Direction (*F* (1, 35) = 6.413, *p* = 0.016, *η*^2^ = 0.155), indicating that the percentage of fixation count within word AOIs was significantly lower for upright images compared to inverted images (*t*(36) = −2.444, *p* = 0.016, *d* = −1.571). The two-way interaction between Reading Group and Stimulus Category was also significant (*F* (1, 35) = 4.742, *p* = 0.036, *η*^2^ = 0.119). In the low-reading ability group, the percentages of fixation count on both words in environmental print and ordinary words were significantly lower than those in the high-reading ability group (*t*(35) = −2.885, *p* = 0.007, *d* = −4.167 for words in environmental print, (*t*(35) = −3.667, *p* < 0.001, *d* = −5.211 for ordinary words). There was no significant difference between words in environmental print and ordinary words in the low-reading ability group (*t*(17) = −0.750, *p* = 0.440, *d* = −0.316). However, for children with high reading ability, the percentages of fixation count on words in environmental print was significantly lower than on ordinary words (*t*(18) = −2.250, *p* = 0.026, *d* = −0.947). Importantly, the three-way interaction effect among Reading Group, Word Direction, and Stimulus Category was also significant (*F* (1, 35) = 5.813, *p* = 0.021, *η*^2^ = 0.142). Similar to the results for fixation duration, only children in the high-reading ability group showed higher percentage of fixation count in word AOIs when viewing logos with words in environmental print in the upright condition (*t*(18) = 2.933, *p* = 0.007, *d* = 2.200).

**Figure 4:**
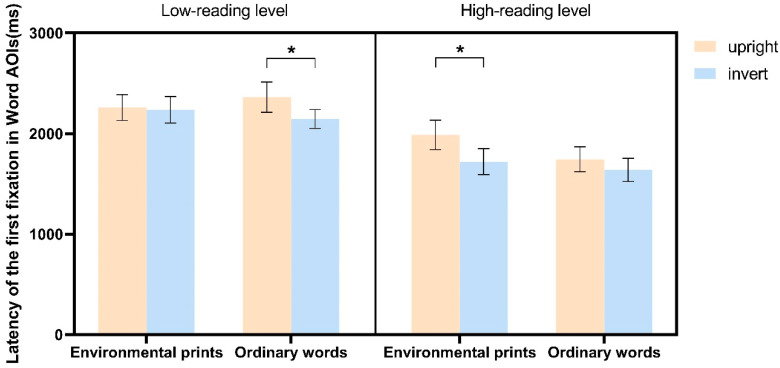
The latency of the first fixation in word AOIs across four stimulus categories in children at different levels of reading ability. Error bars denote ± standard error of measurement. * *p* < 0.05.

Figure 4 illustrated *the latency of the first fixation in word AOIs* for the two groups of children. Results revealed a significant main effect of Word Direction (*F* (1, 35) = 8.409, *p* = 0.006, *η*^2^ = 0.194), indicating that the first fixation latency in the word AOIs was significantly longer for upright images than for inverted images. The main effect of Stimulus Category was marginally significant (*F* (1, 35) = 3.723, *p* = 0.062, *η*^2^ = 0.096), with words in environmental print showing a longer latency of the first fixation in the word AOIs compared to ordinary words (*t*(36) = 1.929, *p* = 0.062, *d* = 0.963). The two-way interaction between Reading Group and Stimulus Category was significant (*F* (1, 35) = 4.309, *p* = 0.045, *η*^2^ = 0.110). The first fixation latency in word AOIs for children in the low-reading ability group was significantly longer than for children in the high-reading ability group, both for words in environmental print (*t*(35) = 2.257, *p* = 0.030, *d* = 3.389) and ordinary words (*t*(35) = 3.465, *p* = 0.001, *d* = 4.967). There was no significant difference between words in environmental print and ordinary words in the low-reading ability group (*t*(17) = −0.102, *p* = 0.919, *d* = 0.051), However, in the high-reading ability group, the first fixation latency for words in environmental print was significantly greater than for ordinary words (*t*(18) = 2.871, *p* = 0.007, *d* = 1.433). Importantly, the three-way interaction effect among Reading Group, Word Direction, and Stimulus Category was significant (*F* (1, 35) = 4.471, *p* = 0.042, *η*^2^ = 0.113). As depicted in Figure 4, among children in low-reading level group, no distinction was observed in the first fixation latency in word AOIs for words in environmental print between inverted and upright images (*t*(17) = 2.871, *p* = 0.833, *d* = 0.158). However, for ordinary words, the first fixation latency for upright images was significantly longer than for inverted images (*t*(17) = 2.447, *p* = 0.020, *d* = 1.559). In contrast, among children in high-reading level group, their first fixation latency in word AOIs for words in environmental print differed between inverted and upright images, with a significantly longer latency for upright images (*t*(18) = 2.632, *p* = 0.013, *d* = 1.953). For ordinary words, however, there was no such difference (*t*(18) = 1.221, *p* = 0.230, *d* = 0.778).

## 9. Discussion

The main goal of our study was to investigate the impact of children’s reading abilities on their attention to inversion sensitivity to words in environmental print during the early stages of reading development. Using eye-tracking technology combined with a grouping design, we found that, in terms of fixation frequency and durations, only children with higher reading ability demonstrated inversion sensitivity to words in environmental print. No significant inversion effect was observed in children with lower reading abilities. Additionally, regarding the latency of the first fixation in word AOIs, children with higher reading ability exhibited inversion sensitivity to words in environmental print, whereas children with lower reading abilities displayed inversion sensitivity to ordinary words.

Consistent with previous studies [8,9,10], we observed inversion sensitivity to words in preschool children. Our results showed that the latency of the first fixation on inverted words was shorter than that for upright words. This early indicator of attentional focus, denoted by the first fixation latency, reflects the speed at which subjects redirect their attention to a specific area of interest (AOI) upon stimulus presentation [37,38]. Our findings suggest that preschool children orient their attention more quickly to inverted words. In addition, we observed that both the percentage of fixation duration and the number of fixations for inverted words were greater than for upright words. These measures of sustained attention—fixation duration and count—represent an individual’s ability to maintain attention on a target over time [38]. Therefore, our results suggest that inverted words sustained children’s attention more. Previous research has highlighted differences in reaction times between inverted and upright words in visual word-matching tasks, demonstrating children’s inversion sensitivity from the perspective of visual perception [10]. Our results extend previous studies by showing that preschool children also exhibit inversion sensitivity to words in terms of attentional processes.

Our fist main finding was the difference in children’s inversion sensitivity to words in environmental print compared to ordinary words. As shown by the percentage of fixation durations, when children were presented with words in environmental print, the fixation durations for upright words were notably shorter than those for inverted words, whereas no such difference was observed for ordinary words. One possible explanation for this finding relates to the exposure children have to environmental print in their daily surroundings [31]. Environmental print—such as logos, brand names, and public signage—is pervasive in children’s environments, and they tend to show an attentional bias towards these words due to their frequent occurrence [5,15,39]. These familiar words are a significant part of early literacy development [17]. Consequently, children are more accustomed to upright words in environmental print than ordinary words. This finding aligns with previous research suggesting that visual familiarity plays a key role in influencing children’s inversion sensitivity to words. Specifically, children tend to exhibit inversion sensitivity to high-familiarity words, while low-familiarity words do not elicit significant inversion sensitivity [9]. Therefore, children showed inversion sensitivity to words in environmental print.

The second and most important finding of our study is that children with higher reading abilities demonstrated inversion sensitivity when viewing words in environmental print, whereas children with lower reading abilities did not exhibit significant inversion sensitivity. These results underscore substantial inter-individual differences in inversion sensitivity to words during the early stage of reading development. Consistent with our findings, previous studies have shown that inversion sensitivity to ordinary words is influenced by children’s reading abilities [8]. This research indicated a significant correlation between Chinese character inversion sensitivity and reading ability. Since words in environmental print are also a form of visual words, it follows that inversion sensitivity to words in environmental print would similarly be affected by reading ability. Consequently, we observed inversion sensitivity to words in environmental print only in children with higher reading ability. Additionally, while previous studies have examined the relationship between inversion sensitivity to words and reading ability across different age groups [9,10], our research highlights that even among peers of the same age, there are significant individual differences in inversion sensitivity to words in children with low and high reading abilities.

Unexpectedly, the results of the first fixation latency are not entirely inconsistent with the above conclusions. Children in the lower reading level group also exhibited an inversion sensitivity effect toward ordinary words in the latency of the first fixation in word AOIs. We believe these results may be related to the relationship between visual familiarity and inversion sensitivity, which may follow an inverted U-shaped pattern rather than a linear one [40]. Specifically, inversion sensitivity is associated with words that are either highly or minimally familiar, but not with those of moderate familiarity. For children with low reading ability, words in environmental print are moderately familiar, whereas ordinary words are entirely unfamiliar stimuli. Therefore, they exhibit inversion sensitivity to ordinary words. In contrast, for children with high reading ability, ordinary words are moderately familiar, while words in environmental print are highly familiar stimuli. Consequently, they display an inversion sensitivity to words in environmental print but not ordinary words. However, the mechanism underlying these unexpected results remains an important and open question for future investigations. Future studies should employ a broader range of words with varying levels of visual familiarity (high, moderate, and low).

Before closing our discussion, we would like to note some limitations of our study. First, we used a 19-inch monitor to present the stimuli. In the future, employing a mobile eye tracker to record children’s eye movements toward environmental words in real-life settings may yield more natural and intuitive results. Second, we used a grouping design to examine the effect of reading ability on inversion sensitivity to words in environmental print. It remains uncertain whether there is a causal link between children’s inversion sensitivity to words in environmental print and their reading ability. Further longitudinal follow-up studies may provide evidence for whether reading ability can modulate the development of children’s inversion sensitivity to words in the environment. Third, we only examined children aged 4 to 5 years. Therefore, we have little knowledge about whether the observed effect of reading ability on attention to the inversion of words in environmental print persists as children continue to grow and develop. Future studies including children of diverse ages may help us further understand how such effects develop with age increases. Another point is that the familiarity of the stimulus materials requires further consideration. It remains unclear whether differences in children’s attention still exist when comparing words in environmental print to simple, common words that are more familiar to preschoolers. Future research needs to compare words in environmental print with Chinese characters of varying degrees of visual familiarity/frequency to further examine the role of environmental print experiences.

In summary, we demonstrated heightened sensitivity to the inversion of environmental words compared to ordinary words. Children with high reading abilities exhibit inversion sensitivity to words in environmental print, whereas those with low reading abilities did not show significant sensitivity. Our study used eye movement technology to provide more evidence in reinforcing the importance of environmental print experience in children’s early literacy development from the perspective of print awareness. It also extends our understanding of the development of inversion sensitivity during children’s early reading development. Based on our findings, we propose several suggestions for incorporating words in environmental print into early literacy education. First, preschool teachers can incorporate more interactive activities with environmental print, such as interesting games, asking questions, and using finger reading strategies to enhance children’s interaction with words in environmental print [41]. Second, teachers and parents should enrich children’s daily environment with environmental print. Such exposure to words in environmental print may enhance children’s awareness of Chinese characters and facilitate their print awareness, including understanding word orientation.

## Figures and Tables

**Table 1:** Participants’ information and their reading performance.

Group	N (Male)	Age	Reading Score
Low-level reading	18 (8)	5.06 (0.24)	6.22 (3.90)
High-level reading	19 (10)	5.00 (0.00)	27.42 (15.44)

Notes: SD of the mean is given in parentheses.

## Data Availability

The data supporting this study’s findings are available on request from the corresponding author. The data are not publicly available due to privacy or ethical restrictions.
